# Case report: Novel compound heterozygous *IL1RN* mutations as the likely cause of a lethal form of deficiency of interleukin-1 receptor antagonist

**DOI:** 10.3389/fimmu.2024.1381447

**Published:** 2024-04-05

**Authors:** Elena Urbaneja, Nuria Bonet, Manuel Solis-Moruno, Anna Mensa-Vilaro, Iñaki Ortiz de Landazuri, Marc Tormo, Rocio Lara, Susana Plaza, Virginia Fabregat, Jordi Yagüe, Ferran Casals, Juan I. Arostegui

**Affiliations:** ^1^ Department of Immunology and Pediatric Rheumatology, Hospital Clínico Universitario de Valladolid, Valladolid, Spain; ^2^ Genomics Core Facility, Departament de Medicina i Ciències de la Vida (MELIS), Universitat Pompeu Fabra, Parc de Recerca Biomèdica de Barcelona, Barcelona, Spain; ^3^ Department of Immunology, Hospital Clínic, Barcelona, Spain; ^4^ Institut d’Investigacions Biomèdiques August Pi i Sunyer, Barcelona, Spain; ^5^ Scientific Computing Core Facility, Departament de Medicina i Ciències de la Vida (MELIS), Universitat Pompeu Fabra, Parc de Recerca Biomèdica de Barcelona, Barcelona, Spain; ^6^ School of Medicine, Universitat de Barcelona, Barcelona, Spain; ^7^ Departament de Genètica, Microbiologia i Estadística, Facultat de Biologia, Universitat de Barcelona, Barcelona, Spain; ^8^ Institut de Biomedicina de la Universitat de Barcelona (IBUB), Universitat de Barcelona, Barcelona, Spain; ^9^ Centro de Investigaciones Biomédicas en Red de Enfermedades Raras (CIBERER), Instituto de Salud Carlos III (ISCIII), Madrid, Spain

**Keywords:** case report, interleukin-1, interleukin-1 receptor antagonist (IL-1 ra), autoinflammatory diseases (AID), whole-genome sequencing (WGS)

## Abstract

Undiagnosed monogenic diseases represent a challenging group of human conditions highly suspicious to have a genetic origin, but without conclusive evidences about it. We identified two brothers born prematurely from a non-consanguineous healthy couple, with a neonatal-onset, chronic disease characterized by severe skin and bone inflammatory manifestations and a fatal outcome in infancy. We conducted DNA and mRNA analyses in the patients’ healthy relatives to identify the genetic cause of the patients’ disease. DNA analyses were performed by both Sanger and next-generation sequencing, which identified two novel heterozygous *IL1RN* variants: the intronic c.318 + 2T>G variant in the father and a ≈2,600-bp intragenic deletion in the mother. *IL1RN* mRNA production was markedly decreased in both progenitors when compared with healthy subjects. The mRNA sequencing performed in each parent identified two novel, truncated *IL1RN* transcripts. Additional experiments revealed a perfect intrafamilial phenotype–genotype segregation following an autosomal recessive inheritance pattern. The evidences shown here supported for the presence of two novel *loss-of-function* (LoF) *IL1RN* pathogenic variants in the analyzed family. Biallelic LoF variants at the *IL1RN* gene cause the deficiency of interleukin-1 receptor antagonist (DIRA), a monogenic autoinflammatory disease with marked similarities with the patients described here. Despite the non-availability of the patients’ samples representing the main limitation of this study, the collected evidences strongly suggest that the patients described here suffered from a lethal form of DIRA likely due to a compound heterozygous genotype at *IL1RN*, thus providing a reliable genetic diagnosis based on the integration of old medical information with currently obtained genetic data.

## Introduction

Autoinflammatory diseases (AIDs) represent a special group among inborn errors of immunity (IEI) since they are characterized by recurrent episodes of sterile inflammation, with no increased risk to severe infections (1). Over the past 20 years, ≈60 different monogenic AIDs have been identified, and different successful treatments have been reported (1, 2). The deficiency of interleukin-1 (IL-1) receptor antagonist (IL-1Ra), abbreviated as DIRA, is a rare monogenic AID, with less than 25 families described worldwide, provoked by biallelic *loss-of-function* variants at *IL1RN*. These variants typically lead to the loss of IL-1Ra production, the endogenous inhibitor of the proinflammatory cytokines IL-1α and IL-1β (3, 4). DIRA starts typically during early infancy with sterile inflammation mainly affecting skin, joints, and bones. Daily treatment with anakinra, the recombinant form of human IL-1Ra, resulted in terrific clinical responses and normalization of laboratory parameters (3, 4).

Herein we describe an undiagnosed and devastating inflammatory disease observed in two siblings born in 1970–1980s from a non-consanguineous healthy couple. Genetic investigations performed four decades later identified two novel *IL1RN* variants in their family, and additional studies demonstrated their deleterious consequences at the *IL1RN* mRNA production by different mechanisms. These evidences strongly suggest that the patients herein described probably suffered from DIRA as a consequence of a compound heterozygous genotype at *IL1RN* and died as a consequence of the natural course of the disease. We also discussed the limitations of our study to unequivocally establish DIRA as their definitive diagnosis mainly due to the non-availability of the patients’ samples.

## Subjects and methods

### Subjects

The patients’ clinical data and laboratory results were collected from medical charts. Written informed consents to participate in the study and to publish the results were obtained from each enrolled alive individual. The Ethical Review Board of Hospital Clínic, Spain approved the study (code HCB/2022/0855). All investigations were performed in accordance with the ethical standards of the 1964 Declaration of Helsinki and its later amendments.

### DNA analyses

DNA samples were prepared from peripheral blood using QIAmp DNA Blood Mini Kit (QIAgen, Germany). The next-generation sequencing methods employed included a targeted gene panel (TGP; **Supplementary Table S1**) and whole genome sequencing (WGS). TGP was generated in an Access Array System 48.48 platform (Fluidigm, USA), whereas WGS was prepared using the lllumina DNA PCR-Free Library Prep kit (Illumina, USA). Library preparation and control quality were performed according to the manufacturers’ instructions, and sequencing was performed on NextSeq or NovaSeq 6000 platforms (Illumina, USA). Reads were mapped against the GRCh37 using the BWA software (5), and their analyses were performed using the SeqNext software (JSI Medical Systems, Germany). Detected variants were classified according to previously published recommendations (6).

For the Sanger method of DNA sequencing, amplicons of *IL1RN* gene (RefSeq NM_173842.3) were in-house designed and PCR-amplified, purified with Illustra ExoStar 1-Step kit (GE Healthcare, USA), bidirectionally sequenced using ABI BigDye^®^ Terminator v3.1 Cycle Sequencing Kit (Applied Biosystems, USA), and run on the ABI 3730XL DNA analyzer (Applied Biosystems, USA). Reads analysis was performed using the SeqPilot software (JSI Medical Systems, Germany).

### RNA analyses

RNA samples from family members and healthy individuals were prepared from peripheral blood collected in Tempus™ tubes (Thermo Fisher Scientific, USA). To evaluate *IL1RN* mRNA production, cDNA was generated by the Superscript II reagent kit (Invitrogen, USA). Quantitative polymerase chain reaction (qPCR) was performed using two different TaqMan (Thermo Fisher Scientific, USA) probes for *IL1RN*: CustomEx1 probe, targeting exon1 of *IL1RN*, and Hs00893626_m1, targeting a region covering the end of exon 3 and the beginning of exon 4 of *IL1RN*, on a QuantStudio 12K Flex Real-Time PCR System (Thermo Fisher Scientific, USA). Gene expression levels were calculated according to the ΔΔC_t_ method and normalized against two endogenous controls (*GAPDH*, probe Hs0786624_g1; *ACTB*, probe Hs01060665_g1; Thermo Fisher Scientific, USA).

To characterize the sequence of the different *IL1RN* transcripts, RNA libraries were prepared from total mRNA using NEBNext Ultra II Directional RNA Library Prep Kit for Illumina (New England Biolabs, USA) with the rRNA depletion configuration. RNA libraries were validated using TapeStation (Agilent Technologies, USA), pooled and quantified by qPCR, and sequenced on a NextSeq platform (Illumina, USA) using the NextSeq 500 High-Output 150-paired-end 2 × 75 cycles kit (Illumina Inc, USA). Sequence reads were aligned to GRCh38 using the Ensembl annotation version 109 with STAR (version 2.7.0d). Gene expression quantification at the exon level and differential exon usage analysis were performed using the DEXSeq package in R. To identify novel transcripts, the sequence reads were aligned to the same reference using Bowtie2 (version 2.3.4.1) and Tophat (version 2.1.1). Subsequently, we employed specific applications from Cufflinks (v2.2.1) for further analysis. Initially, BAM files obtained from previous steps were filtered based on our region of interest. Cuffcompare was used to predict transcripts for each group, and gffread was utilized to generate the FASTA files of the predicted transcripts.

## Results

### Patients

**Figure 1:**
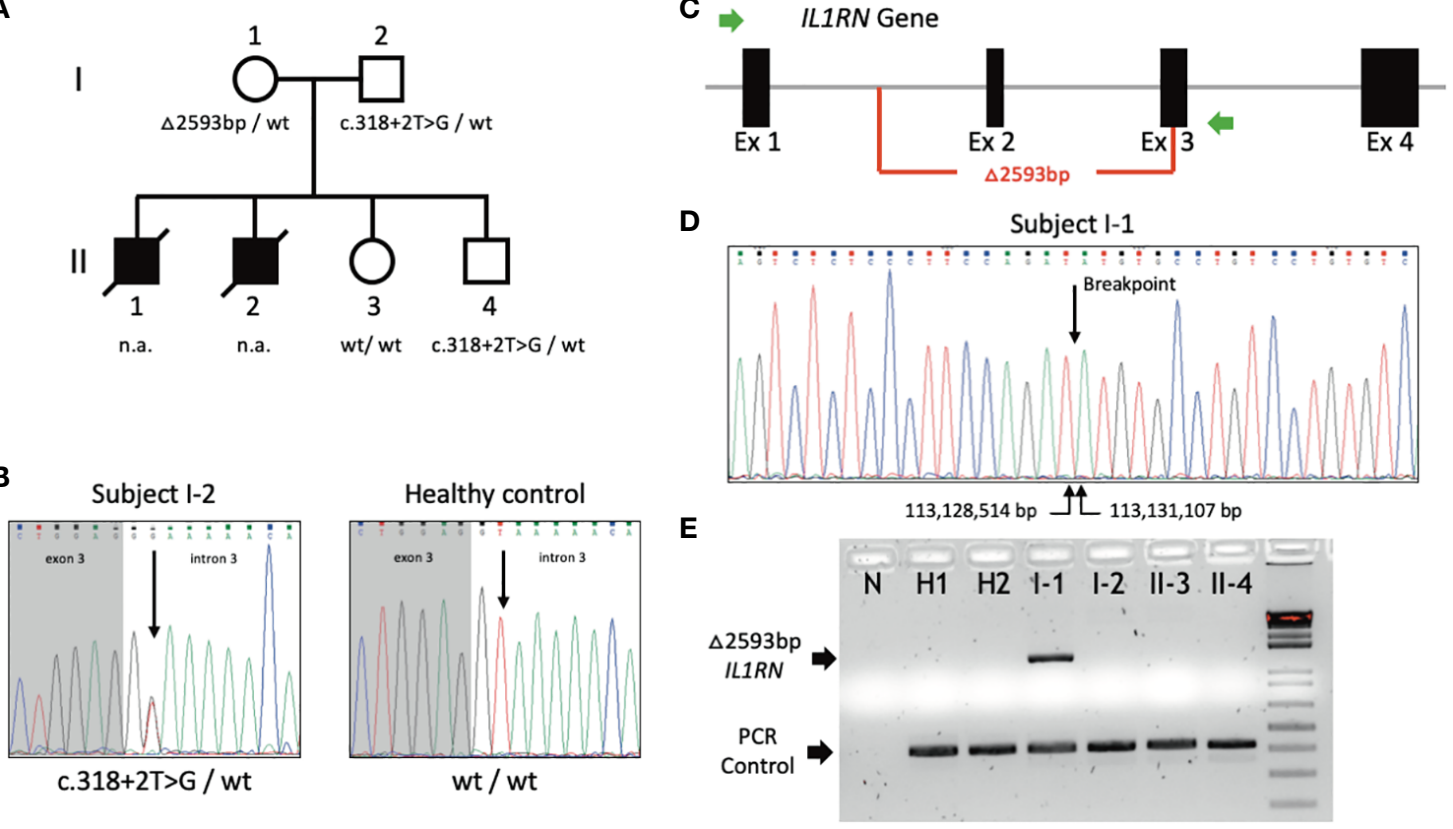
*IL1RN* variants detected in enrolled individuals. **(A)** Pedigree of the family. Black-filled symbols, affected subjects; open symbols, unaffected subjects; squares, male subjects; circles, female subjects; slash, deceased subjects. *IL1RN* genotypes are shown below each analyzed subject. n.a., not analyzed; wt, wild-type. **(B)** Sense Sanger chromatograms from subject I-2 carrying the heterozygous genotype for the c.318 + 2T>G *IL1RN* variant (left box) and from a healthy subject (right box). The arrows indicate the position where the nucleotide variant is located. **(C)** Genomic organization of isoform 1 of *IL1RN* gene (NM_173842.3). Green arrows represent the forward and reverse primers designed to generate a PCR amplicon specific of the genomic deletion. **(D)** Sense Sanger chromatogram showing the breakpoint and boundaries of the genomic deletion at *IL1RN* locus identified in subject I-1. The arrows indicate the nucleotides located at each side of the breakpoint site, and shown below are the respective genomic coordinates according to GRCh38. **(E)** Agarose gel electrophoresis of PCR products generated with the use of primers designed for genomic deletion. N, negative control; H1 and H2, healthy subjects. Black arrows indicate the specific bands of PCR amplicons of the *IL1RN* allele containing the deletion (top) and a positive control of PCR reaction (bottom).

We identified a non-consanguineous healthy couple of Spanish ancestry with four children, two of which were affected by an undiagnosed and lethal inflammatory disease (**Figure 1A**). One of these patients (patient II-1) was previously reported (7). Both patients were born prematurely at 36 weeks of gestation in 1977 and 1983, respectively. Their disease started during the first week of life, with similar clinical manifestations and laboratory perturbations (**Table 1**). The disease had a chronic course and a fatal outcome in both cases: patient II-1 died at the age of 13 months as a consequence of a complicated varicella infection, whereas patient II-2 died at the age of 3 years due to a cerebral thrombosis.

**Table 1:** Clinical manifestations and genetic data of enrolled patients and reported patients with DIRA. .

		Patient II-1	Patient II-2	Reported DIRA patients (*n* = 28)
Sex		Male	Male	15 male/13 female
Gestational age^a^	Premature	Yes (36 weeks)	Yes (36 weeks)	88.9% (16/18)
	At term	–	–	11.1% (2/18)
Age at disease onset^b^	0–1 month	Yes	Yes	69.2% (18/26)
	2–6 months	–	–	15.4% (4/26)
	>6 months	–	–	15.4% (4/26)
Clinical outcome	Alive	No	No	78.6% (22/28)
	Deceased	Yes	Yes	21.4% (6/28)
	Age at death^c^	13 months	3 years	12.5 months (range: intra-uterus–9.5 years)
	Cause of death	Varicella infection	Cerebral thrombosis	Multiorgan failure secondary to SIRS (*n* = 2) Pulmonary hemosiderosis (*n* = 1) Medical pregnancy interruption (*n* = 1) CMV-induced hemophagocytosis (*n* = 1) Not specified (n:1)
Fever		Yes	Yes	34.6% (9/26)
Pustulosis		Yes	Yes	92.3% (24/26)
Musculo-skeletal manifestations	Arthritis	No	No	53.8% (14/26)
	Sterile multiple osteolytic lesions	No	No	74.1% (20/27)
	Ribs involvement	Yes	Yes	67.8% (19/28)
	Clavicles involvement	Yes	Yes	21.0% (4/19)
	Long bones involvement	Yes	Yes	Not systematically specified
	Periosteal elevations	Yes	Yes	73.1% (19/26)
Hepatomegaly		No	No	26.3% (5/19)
Splenomegaly		No	No	26.3% (5/19)
Irritability		Yes	Yes	Not specified
Sleep disturbance		Yes	Yes	Not specified
Conjunctivitis		Yes	Yes	15.4% (4/26)
Lung disease		No	No	19.2% (5/26)
Thrombosis		No	Yes	26.7% (4/15)
Other manifestations	Growth retardation	Yes	Yes	Common, but not systematically specified
	Hypotonia	Yes	Yes	Occasionally reported
	Recurrent Infections	Yes	Yes	15.4% (4/26)
	Cardiac Insufficiency	Yes	Yes	Not specified
Genetics				
*IL1RN* Genotype^d^	Homozygous	No	No	91.3% (21/23)
	Compound Heterozygous	Yes	Yes	8.7% (2/23)
*IL1RN* variants^e^	Missense	No	No	None
	Nonsense	No	No	*n* = 7 S21X (*n* = 2), R26X (*n* = 2), R29X (*n* = 2), Q45X (*n* = 1), Q54X (*n* = 6), E77X (*n* = 11), Q119X (*n* = 4)
	Frameshift deletion	No	No	*n* = 6 N18KfsX4 (*n* = 4), T47TfsX4 (*n* = 1), N52KfsX25 (*n* = 2), D72_I76 del (*n* = 6), T133PfsX118 (*n* = 2)
	Splice site	Yes	Yes	None
	Large genomic deletions	Yes	Yes	*n* = 2 22.2kb del (*n* = 2), 175 kb del (*n* = 8)

SIRS, severe inflammatory response syndrome; CMV, cytomegalovirus.

a Data available for 18 patients.

b Data available for 26 patients.

c Median age at death of reported DIRA patients.

d IL1RN genotypes identified in the 23 different families reported as suffering from DIRA.

e IL1RN variants identified in the reported DIRA families. The number of alleles with the IL1RN variants is shown among brackets.

Their main clinical features included recurrent fever and skin and musculoskeletal manifestations. Skin lesions were characterized by generalized, severe vesiculo-pustular lesions, often over-infected, refractory to all administered treatments. Musculoskeletal manifestations included marked pain with movement and painful swelling at clavicles, ribs, femur, and mandibula. The radiographic findings included periosteal elevations of long bones and widening of clavicles and multiple ribs. On the basis of these lesions, patient II-1 was diagnosed as having Caffey–Silverman syndrome, and corticosteroid treatment led to a transient relief. Additional manifestations included hypotonia, irritability, marked development and growth retardation, conjunctivitis, aseptic meningitis, hydrocephaly, cardiac insufficiency, and a tendency to mild-to-moderate infections (oral thrush, otitis, urinary infection, and gastroenteritis).

The results of laboratory tests revealed increased leukocyte (20.1–55.5 × 10^9^/L; reference range [RR] 6–17 × 10^9^/L) and platelet (620 × 10^9^/L; RR 150–450 ×10^9^/L) counts, decreased hemoglobin concentration (7.4–10.7 g/dL; RR 10.7–14.7 g/dL), and a marked increase of erythrocyte sedimentation rate (>75 mm/h; RR <10 mm/h). All analyses evaluating the immune system performed at that time (complement, circulating immunoglobulins, oxidative burst test, PHA-stimulated lymphocytic test, PPD test, and thymic shadow) were normal or negative (7).

### DNA analyses

On the basis of the patients’ features and familial pedigree, we hypothesized for the presence of an early-onset, severe monogenic inflammatory disease inherited either as a recessive or as an X-linked trait. As biological samples from patients were not currently available, we genetically analyzed the patients’ first-degree healthy relatives (see **Figure 1A** to identify the analyzed individuals). We first performed a screening of monogenic AID-associated genes using a TGP, which detected in the patients’ father the heterozygous c.318 + 2T>G transversion in the donor splice site at intron 3 of *IL1RN* (**Figure 1B**). This variant has not been previously reported, and bioinformatics analyses suggested that it may impair the normal mRNA production (**Supplementary Table S2**).

DIRA diagnosis might fit with the patients’ clinical features, but the absence of *IL1RN* variants in the patients’ mother apparently excluded a confirmatory genotype for this recessively inherited disease. Considering the intrinsic limitations of the methods employed thus far, we performed additional analyses in the patients’ mother to identify a potential second hit at *IL1RN*. WGS analysis was performed in a DNA sample from the patients’ mother, which revealed a heterozygous intragenic *IL1RN* deletion (**Supplementary Figure S1**). A specific PCR with primers at each side of the breakpoint was designed (**Figure 1C**), and the amplicon sequencing revealed a 2,593-bp deletion encompassing from intron 1 until exon 3 (**Figure 1D**, **Supplementary Table S** **2** and **Supplementary Figure S2**). This genomic deletion has not been reported previously, and neither has it been registered in public databases. Intrafamilial segregation of *IL1RN* variants revealed that the patients’ first-degree healthy relatives carried either a single heterozygous *IL1RN* genotype (each of the patients’ parent and their brother) or a homozygous genotype for the wild-type allele (patients’ sister), thus confirming that none of them carried a confirmatory genotype for DIRA (**Figures 1A, E**).

### mRNA analyses

**Figure 2:**
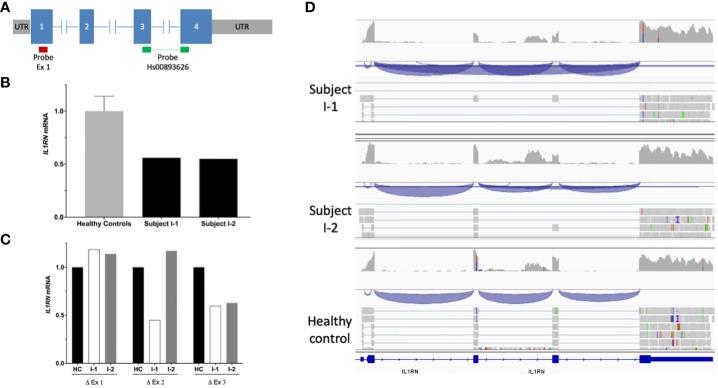
| mRNA *IL1RN* analysis. **(A)** Scheme of *IL1RN* gene (isoform 1; RefSeq: NM_173842.3) and location of probes employed in the quantitative PCR assay. The red bar indicates the probe mapping in exon 1, whereas the green bars indicate the probe Hs00893626, which maps in the junction of exons 3 and 4. **(B)** Relative mRNA levels of *IL1RN* in the peripheral blood of healthy subjects (*n* = 8) and patients’ parents determined by quantitative PCR. The bars indicate the ratio of exon 3 *versus* exon 1, while T bars indicate the standard deviations, where applicable. **(C)** Relative quantification of exons of *IL1RN* determined by mRNA sequencing. The bars indicate the mean of the fold change among parents and healthy controls. **(D)** Schemes of novel *IL1RN* mRNA transcripts showing the exon junctions (blue) identified by mRNAseq in subject I-1 (upper panel) and subject I-2 (middle panel) compared with the normal mRNA transcripts identified in a healthy subject (bottom panel).

Prediction analyses suggested that the novel *IL1RN* variants detected here may provoke a decrease in mRNA production by different mechanisms. The intronic variant is predicted to impair the normal splicing of immature mRNA by destroying one of the canonical splice sites of the gene, whereas the allele containing the genomic deletion is predicted to produce a mRNA transcript smaller than that encoded by the wild-type allele (534 bp in wild-type allele vs. 383 bp in mutant allele). We first evaluated the production of wild-type *IL1RN* mRNA transcript by using qPCR analyses (**Figure 2A**), which revealed a marked decrease of production in each of the patients’ progenitor when compared to healthy subjects (**Figure 2B**), a result that was subsequently validated by mRNA sequencing (**Figure 2C**).

To investigate the consequences at the molecular level of the novel *IL1RN* variants, we performed mRNA sequencing in each of the patients’ parent. These analyses identified two novel *IL1RN* transcripts predicted to generate truncated IL-1Ra proteins. On one side, the allele containing the c.318 + 2T>G intronic variant generated a novel transcript with the skipping of exon 3 (**Figure 2D**), which caused a frameshift mutation, the incorporation of two aberrant amino acid residues, and a premature stop codon (p.Glu69Glyfs*2) (**Supplementary Figure S3**). On the other side, the allele containing the genomic deletion generated a transcript that lacks exons 2 and 3 of the gene (**Figure 2D**), which also caused a frameshift, the incorporation of six aberrant amino acid residues, and the appearance of a premature stop codon (p.Ile40Glnfs*6) (**Supplementary Figure S4**). All these experimental evidences confirmed that the two novel *IL1RN* variants were *loss-of-function*.

## Discussion

In the genetic counseling consult, we identified a lethal inflammatory undiagnosed disease in two siblings who were born 40 years ago from a healthy, non-consanguineous couple. Their familial and clinical data strongly suggested a genetic cause for the disease, being the autosomal recessive and the X-linked modes of inheritance as the most probable. By using different methods of genetic study, we finally identified two novel *IL1RN* variants in this family. Their *loss-of function* behavior was confirmed by different experiments that revealed both a marked reduction of mRNA production by the variant *IL1RN* alleles and the identification of novel transcripts predicted to generate truncated proteins. Considering all collected clinical, laboratory, and genetic evidences, we propose that the most likely option is that both patients suffered from a lethal form of DIRA as a consequence of a compound heterozygous genotype at *IL1RN*. However, the main limitation of this study is the non-availability of the patients’ samples due to their death 40 years ago. In a strict sense, this limitation prevented us to unequivocally establish DIRA as their definitive diagnosis despite either clinical manifestations or genetic findings strongly pointing toward it.

Since its description, DIRA has been reported in 23 families of different ethnic backgrounds (3, 4, 8–11). From a clinical perspective, DIRA starts often during neonatal period (≈70%), and sterile inflammatory manifestations involving the musculoskeletal system (100%) and skin (92%) represent their hallmarks (**Table 1**). The manifestations of the patients here described are in concordance with those previously reported in DIRA, including some occasional features such as a tendency to develop thrombosis (patient II-2) and mild-to-moderate infections (2, 8). From a genetic point of view, 91% of patients with DIRA carried homozygous genotypes as a consequence of parental consanguinity, with only two patients being a consequence of compound heterozygous *IL1RN* genotypes (**Table 1**). Among all reported *IL1RN* variants causing DIRA, seven variants were single nucleotide exchanges leading to nonsense variants, six were small base pair deletions leading to mRNA frameshift and premature stop codons, and two were large genomic deletions (3, 4, 8–11). The family described here would represent the third known family with DIRA due to a compound heterozygous *IL1RN* genotype, with the additional novelties of the characterization of the first pathogenic variant located in a splice site in the *IL1RN*, and a novel structural variant (intragenic deletion), whose identification required the combination of different techniques.

From a pathophysiological perspective, DIRA represents one of the monogenic AIDs closely related with an increased signaling through the IL-1 pathway (12). The prototypic agonist cytokines driving inflammation in this pathway are called IL-1α and IL-1β. Despite the differences in their amino acid sequences and post-translational modifications, both cytokines bind to the same receptor, the IL-1 receptor type 1 (IL-1R1), and recruit the IL-1R accessory protein (IL-1RAcp) to transduce an intracellular signal (13). In contrast, the protein called IL-1Ra also binds to IL-1R1, but it does not recruit the IL-1RAcP nor transduces an inflammatory signal. As a consequence, IL1Ra competitively antagonizes the pro-inflammatory action of IL-1α and IL-1β (13). The relevance of IL-1Ra has been shown by the description of its genetically determined deficiency in mice and humans (3, 4, 14) as well as by the terrific effectiveness of the treatment with the recombinant IL-1Ra in different human diseases including DIRA (3, 4, 8–11, 15–19). At present, different monogenic AIDs have been associated with an overall increase signaling through the IL-1 pathway by different mechanisms. The cryopyrin-associated periodic syndromes (CAPS) represent the prototypic IL-1-mediated disease, and the IL-1β overproduction is a consequence of *gain-of-function NLRP3* variants that ultimately provoke a hyperactivation of the Nlrp3-inflammasome (20, 21). Patients with CAPS exhibit extremely positive responses to all commercially available IL-1 inhibitors, including the blockade of IL-1α and IL-1β and the endogenous IL-1Ra at the IL-1R1 level (anakinra) (15–17) or in the extracellular space (rilonacept) (22, 23) or by using a monoclonal antibody (MoAb) that specifically binds IL-1β in the extracellular milieu (canakinumab) (24, 25). In the recessively inherited DIRA, the increase of the IL-1 signaling pathway is due to the complete loss of the endogenous inhibitor IL-1Ra due to biallelic *loss-of-function IL1RN* variants (3, 4). Interestingly, most patients with DIRA have been well controlled when treated with the recombinant IL-1Ra anakinra (3, 4, 8–11, 15–19). This therapeutic approach would have been extremely useful in the patients described here. Unfortunately, they were born at a time when IL-1Ra was not yet identified. Finally, a dominantly inherited AID named Loss of IL-1R1 Sensitivity to IL-1Ra (LIRSA) has been recently described as a consequence of monoallelic variants at *IL-1R1* (26). In this disease, the mutation characteristically disrupts the interaction of IL-1R1 with IL-1Ra. As a consequence, there is a hyperactivation of the IL-1 signaling related with the functional loss of the endogenous IL-1Ra (26). Patients with LIRSA did not exhibit positive responses to the treatment with the recombinant IL-1Ra anakinra on the basis of the defect in the IL-1R1 that made it unsensitive to this protein. In contrast, these patients are well controlled with the anti-IL-1β MoAb canakinumab (26). In addition, novel evidences also suggest that these patients may be potentially well controlled with rilonacept or with the novel IL-1α/IL-1β trap rilabnacept (26) (see **Table 2** for a detailed comparison of these IL-1-mediated diseases).

**Table 2:** Comparative features of IL-1-mediated diseases.

	CAPS	DIRA	LIRSA
Gene	*NLRP3*	*IL1RN*	*IL1R1*
Protein	Cryopyrin	IL-1Ra	IL-1R type 1
Pathophysiological mechanism	*Gain-of-function* variants	*Loss-of-function* variants	Variants causing loss of IL-1R1 sensitivity to IL-1Ra
Inheritance pattern	1. Autosomal dominant (familial cases) 2. *De novo* germline variants (sporadic cases) 3. *De novo* post-zygotic variants (mosaicism; sporadic cases)	Autosomal recessive	*De novo* germline variants (sporadic cases)
Clinical manifestations			
Disease onset	1. Infancy/first decade 2. Occasional reports of adult-onset disease (frequent in mosaicism carriers)	Birth/infancy	First year of life
Recurrent fever	Yes	Frequent (≈ 1/3 of patients)	Not reported
Skin lesions	Generalized urticaria-like skin rash (often triggered by cold exposure)	Generalized pustulosis	Not reported
Articular manifestations	Arthralgias Recurrent arthritis Occasional arthropathy (knees)	Arthralgias Recurrent arthritis Occasional arthropathy (knees) Osteolytic lesions Periosteal elevations Swelling at ribs, clavicles	Arthralgias Recurrent arthritis Chronic pain Osteolytic lesions CRMO
Ocular manifestations	Conjunctivitis Papiledema Occasional uveitis	Occasional conjunctivitis (≈1/5 patients)	Not reported
CNS manifestations	Headache Recurrent aseptic meningitis	Hypotonia Developmental delay	Not reported
Hearing loss	Frequent (≈1/3–1/2 patients)	Not reported	Not reported
AA-amyloidosis	Frequent (≈1/5–1/3 patients)	Not reported	Not reported
Tendency to thrombosis	Not reported	Occasionally reported	Not reported
Response to commercially available IL-1 inhibitors			
Recombinant IL-1Ra (anakinra)	Positive	Positive	No response
IL-1α/IL-1β/IL-1Ra Trap (rilonacept)	Positive	Positive (occasional reports)	Not administered
MoAb anti-IL-1β (canakinumab)	Positive	Positive (occasional reports)	Positive
Number of reported patients	Hundreds	≈25–30	1

CAPS, cryopyrin-associated periodic syndromes; DIRA, deficiency of IL-1 receptor antagonist; LIRSA, loss of IL-1R1 sensitivity to IL-1Ra; IL-1Ra, IL-1 receptor antagonist; IL-1R, IL-1 receptor; CRMO, chronic recurrent multifocal osteomyelitis; CNS, central nervous system.

In all monogenic diseases, the identification of the genetic cause has relevant consequences for the patient in terms of definitive diagnosis and therapeutic management. Moreover, this information may have also consequences for their first-degree relatives due to the moderate-to-high risk of disease recurrence in future pregnancies. This risk of disease recurrence may be present even in those families with undiagnosed diseases but highly suspicious to be monogenic in nature. For all these families, medical advice through an adequate genetic counseling program is highly warranted, even if the patients died many years before as what occurred in the family described here. Indeed this was the scenario in the family analyzed here. We were able to identify and analyze this family until the elucidation of the likely cause of the disease thanks to the request of genetic and medical advices of the patients’ healthy sister in a specific consult, thus concluding a 40-year-long journey since the disease onset until the achievement of the patients’ diagnosis. It is interesting to note that the first results obtained using standard methods of genetic analyses that did not identify the *IL1RN* deletion did not allow to establish the cause of the disease on the basis of a non-confirmatory genotype of DIRA. In similar apparently “negative” cases, a first approach that is being implemented by many laboratories is the re-analysis of previous analyses mainly performed by whole-exome sequencing methods. This approach may have success rates as high as ≈20% mainly thanks to improved bioinformatic pipelines, specific search for gene mosaicisms, reclassification of detected variants, or new gene associations (27, 28). In this work, we describe the possibility of expanding the analysis of particular families by using complementary technologies such as whole-genome sequencing since these methods may increase the power to identify previously undetected variants, especially structural gene variants such as the *IL1RN* deletion described here. Ideally, if the two pathogenic variants at *IL1RN* would have been detected in samples from the two deceased patients, that would represent a post-mortem genetic analysis (29). In a strict sense, this was not possible due to the non-availability of the patients’ samples. However, as a conclusion, the combination of old medical information collected from registries associated with current genetic data obtained in first-degree healthy relatives reasonably provides a reliable diagnosis of DIRA in the analyzed family.

In conclusion, we described two patients who died 40 years ago from an undiagnosed inflammatory disease, of which currently performed studies strongly suggest that they were affected by a lethal form of DIRA. Experimental evidences expand the genetic basis of DIRA by the description of two novel *loss-of-function IL1RN* variants that generate mRNA transcripts predicted to produce truncated proteins. Finally, we highlight the relevance of an adequate genetic counseling in families with undiagnosed but potentially monogenic diseases because this work was possible thanks to the request for medical and genetic advice of the patients’ first-degree healthy relative.

## Data availability statement

The data presented in this study are deposited in the NCBI-BioProject database, accession number PRJNA1092963. http://www.ncbi.nlm.nih.gov/bioproject/1092963.

## Ethics statement

The studies involving humans were approved by Ethical Review Board of Hospital Clínic, Spain (code HCB/2022/0855). The studies were conducted in accordance with the local legislation and institutional requirements. The participants provided their written informed consent to participate in this study. Written informed consent was obtained from the individual(s) for the publication of any potentially identifiable images or data included in this article.

## Author contributions

EU: Conceptualization, Data curation, Formal Analysis, Investigation, Methodology, Writing – review & editing. NB: Conceptualization, Data curation, Investigation, Methodology, Writing – review & editing, Formal Analysis, Software. MS-M: Conceptualization, Data curation, Formal Analysis, Investigation, Methodology, Software, Writing – review & editing. AM-V: Data curation, Formal Analysis, Investigation, Methodology, Writing – review & editing, Funding acquisition. IO: Data curation, Formal Analysis, Investigation, Methodology, Writing – review & editing. MT: Data curation, Formal Analysis, Investigation, Methodology, Writing – review & editing, Software. RL: Data curation, Formal Analysis, Investigation, Methodology, Writing – review & editing, Project administration. SP: Data curation, Formal Analysis, Investigation, Methodology, Project administration, Writing – review & editing. VF: Data curation, Formal Analysis, Investigation, Methodology, Project administration, Writing – review & editing. JY: Data curation, Formal Analysis, Investigation, Methodology, Writing – review & editing. FC: Formal Analysis, Investigation, Methodology, Writing – review & editing, Conceptualization, Funding acquisition, Project administration, Resources, Software, Supervision, Validation. JA: Conceptualization, Formal Analysis, Funding acquisition, Investigation, Methodology, Project administration, Resources, Software, Supervision, Validation, Writing – review & editing, Data curation, Visualization, Writing – original draft.

## References

[1] Aksentijevich I Schnappauf O. Molecular mechanisms of phenotypic variability of monogenic autoinflammatory diseases. Nat Rev Rheumatol. (2021) 17:405–25. doi: 10.1038/s41584-021-00614-1 34035534

[2] Broderick L Hoffman HM. IL-1 and autoinflammatory disease: biology, pathogenesis and therapeutic targeting. Nat Rev Rheumatol. (2022) 18:448–63. doi: 10.1038/s41584-022-00797-1 PMC9210802 35729334

[3] Aksentijevich I Masters SL Ferguson PJ Dancey P Frenkel J van Royen-Kerkhoff A. An autoinflammatory disease with deficiency of the interleukin-1-receptor antagonist. N Engl J Med. (2009) 360:2426–37. doi: 10.1056/NEJMoa0807865 PMC2876877 19494218

[4] Reddy S Jia S Geoffrey R Lorier R Suchi M Broeckel U. An autoinflammatory disease due to homozygous deletion of the IL1RN locus. N Engl J Med. (2009) 360:2438–44. doi: 10.1056/NEJMoa0809568 PMC2803085 19494219

[5] Li H Durbin R. Fast and accurate long-read alignment with Burrows-Wheeler transform. Bioinformatics. (2009) 25(14):1754–60. doi: 10.1093/bioinformatics/btp324 PMC2705234 19451168

[6] Richards S Aziz N Bale S Bick D Das S Gastier-Foster J. Standards and guidelines for the interpretation of sequence variants: a joint consensus recommendation of the American College of Medical Genetics and Genomics and the Association for Molecular Pathology. Genet Med. (2015) 17:405–24. doi: 10.1038/gim.2015.30 PMC4544753 25741868

[7] Blanco A Aragon MP Henales V Merino C Martinez-Robles MV Zurro J. Hiperostosis cortical infantil y varicela fatal. Esp Pediatr. (1981) 14:421–6. 7027854

[8] Ziaee V Youssefian L Faghankhani M Jazayeri A Saeidian AH Vahidnezhad H. Homozygous IL1RN mutation in siblings with deficiency of interleukin-1 receptor antagonist (DIRA). J Clin Immunol. (2020) 40:637–42. doi: 10.1007/s10875-020-00767-w 32170523

[9] Mendonça LO Grossi A Caroli F de Oliveira RA Kalil J Castro FFM. A case report of a novel compound heterozygous mutation in a Brazilian patient with deficiency of Interleukin-1 receptor antagonist (DIRA). Pediatr Rheumatol Online J. (2020) 18:67. doi: 10.1186/s12969-020-00454-5 32819369 PMC7439677

[10] Kuemmerle-Deschner JB Welzel T Hoertnagel K Tsiflikas I Hospach A Liu X. New variant in the IL1RN-gene (DIRA) associated with late-onset, CRMO-like presentation. Rheumatol. (2020) 59:3259–63. doi: 10.1093/rheumatology/keaa119 32259833

[11] Bustamante-Ogando JC Scheffler-Mendoza S Yamazaki-Nakashimada MA Saez-de-Ocariz M. IL-1 receptor antagonist defect (DIRA) in a pediatric patient, receiving adalimumab with good clinical response. Int J Dermatol. (2021) 60(5):639–40. doi: 10.1111/ijd.15411 33426674

[12] Lin B Goldbach-Mansky R. Pathogenic insights from genetic causes of autoinflammatory inflammasomopathies and interferonopathies. J Allergy Clin Immunol. (2022) 149:819–32. doi: 10.1016/j.jaci.2021.10.027 PMC8901451 34893352

[13] Dinarello CA. The IL-1 family of cytokines and receptors in rheumatic diseases. Nat Rev Rheumatol. (2019) 15:612–32. doi: 10.1038/s41584-019-0277-8 31515542

[14] Horai R Saijo S Tanioka H Nakae S Sudo K Okahara A. Development of chronic inflammatory arthropathy resembling rheumatoid arthritis in interleukin 1 receptor antagonist-deficient mice. J Exp Med. (2000) 191:313–20. doi: 10.1084/jem.191.2.313 PMC2195765 10637275

[15] Sibley CH Plass N Snow J Wiggs EA Brewer CC King KA. Sustained response and prevention of damage progression in patients with neonatal-onset multisystem inflammatory disease treated with anakinra: a cohort study to determine three- and five-year outcomes. Arthritis Rheum. (2012) 64:2375–86. doi: 10.1002/art.34409 PMC3474541 22294344

[16] Kuemmerle-Deschner JB Tyrrell PN Koetter I Wittkowski H Bialkowski A Tzaribachev N. Efficacy and safety of anakinra therapy in pediatric and adult patients with the autoinflammatory Muckle-Wells syndrome. Arthritis Rheum. (2011) 63:840–9. doi: 10.1002/art.30149 21360513

[17] Kullenberg T Löfqvist M Leinonen M Goldbach-Mansky R Olivecrona H. Long-term safety profile of anakinra in patients with severe cryopyrin-associated periodic syndromes. Rheumatology. (2016) 55:1499–506. doi: 10.1093/rheumatology/kew208 PMC4957676 27143789

[18] Marko L Shemer A Lidar M Grossman C Druyan A Livneh A. Anakinra for colchicine refractory familial Mediterranean fever: a cohort of 44 patients. Rheumatology. (2021) 60:2878–83. doi: 10.1093/rheumatology/keaa728 34144604

[19] Quartier P Allantaz F Cimaz R Pillet P Messiaen C Bardin C. A multicenter, randomised, double-blind, placebo-controlled trial with the interleukin-1 receptor antagonist anakinra in patients with systemic-onset juvenile idiopathic arthritis (ANAJIS trial). Ann Rheum Dis. (2011) 70:747–54. doi: 10.1136/ard.2010.134254 PMC3070271 21173013

[20] Hoffman HM Mueller JL Broide DH Wanderer AA Kolodner RD. Mutation of a new gene encoding a putative pyrin-like protein causes familial cold autoinflammatory syndrome and Muckle-Wells syndrome. Nat Genet. (2001) 29:301–5. doi: 10.1038/ng756 PMC4322000 11687797

[21] Levy R Gérard L Kuemmerle-Deschner J Lachmann HJ Koné-Paut I Cantarini L. Phenotypic and genotypic characteristics of cryopyrin-associated periodic syndrome: a series of 136 patients from the Eurofever Registry. Ann Rheum Dis. (2015) 74:2043–9. doi: 10.1136/annrheumdis-2013-204991 25038238

[22] Hoffman HM Throne ML Amar NJ Sebai M Kivitz AJ Kavanaugh A. Efficacy and safety of rilonacept (interleukin-1 Trap) in patients with cryopyrin-associated periodic syndromes: results from two sequential placebo-controlled studies. Arthritis Rheum. (2008) 58:2443–52. doi: 10.1002/art.23687 18668535

[23] Hoffman HM Throne ML Amar NJ Cartwright RC Kivitz AJ Soo Y. CAPS-Long-term efficacy and safety profile of rilonacept in the treatment of cryopryin-associated periodic syndromes: results of a 72-week open-label extension study. Clin Ther. (2012) 34:2091–103. doi: 10.1016/j.clinthera.2012.09.009 23031624

[24] De Benedetti F Gattorno M Anton J Ben-Chetrit E Frenkel J Hoffman HM. Canakinumab for the treatment of autoinflammatory recurrent fever syndromes. N Engl J Med. (2018) 378:1908–19. doi: 10.1056/NEJMoa1706314 29768139

[25] Walker UA Tilson HH Hawkins PN Poll TV Noviello S Levy J. Long-term safety and effectiveness of canakinumab therapy in patients with cryopyrin-associated periodic syndrome: results from the β-Confident Registry. RMD Open. (2021) 7:e001663. doi: 10.1136/rmdopen-2021-001663 34001647 PMC8130749

[26] Wang Y Wang J Zheng W Zhang J Wang J Jin T. Identification of an IL-1 receptor mutation driving autoinflammation directs IL-1-targeted drug design. Immunity. (2023) 56:485–501. doi: 10.1016/j.immuni.2023.05.014 37315560

[27] Schobers G Schieving JH Yntema HG Pennings M Pfundt R Derks R. Reanalysis of exome negative patients with rare disease: a pragmatic workflow for diagnostic applications. Genome Med. (2022) 14:66. doi: 10.1186/s13073-022-01069-z 35710456 PMC9204949

[28] Bartolomaeus T Hentschel J Jamra RA Popp B. Re-evaluation and re-analysis of 152 research exomes five years after the initial report reveals clinically relevant changes in 18. Eur J Hum Genet. (2023) 31:1154–64. doi: 10.1038/s41431-023-01425-6 PMC10545662 37460657

[29] Deignan JL De Castro M Horner VL Johnston T Macaya D Maleszewski JJ. Points to consider in the practice of postmortem genetic testing: A statement of the American College of Medical Genetics and Genomics (ACMG). Genet Med. (2023) 25:100017. doi: 10.1016/j.gim.2023.100017 36799919

