# Supplementary material for: Case report: Novel compound heterozygous IL1RN mutations as the likely cause of a lethal form of deficiency of interleukin-1 receptor antagonist
Source: Front Immunol. 2024 Apr 5;15:1381447. doi: 10.3389/fimmu.2024.1381447 (PMC11026629; doi:10.3389/fimmu.2024.1381447)
Supplement: Supplementary file 1 [file DataSheet_1.docx]

**Case Report: Novel Compound Heterozygous *IL1RN* Mutations as the Likely Cause of a Lethal Form of Deficiency of Interleukin-1 Receptor Antagonist**

**Supplementary Appendix**

**Authors**

Elena Urbaneja MD PhD , Nuria Bonet MD PhD, Manuel Solis-Moruno PhD, Anna Mensa-Vilaro PhD, Iñaki Ortiz de Landazuri BsC, Marc Tormo RA, Rocio Lara RA, Susana Plaza RA, Virginia Fabregat RA, Jordi Yagüe MD PhD, Ferran Casals PhD, Juan I. Arostegui MD PhD

**Legends of Supplementary Figures**

**Supplementary Figure S1. Heterozygous *IL1RN* Genomic Deletion Detected by Whole Genome Sequencing.** Mapped reads showing the loss of coverage at *IL1RN* locus in subject I-1 (top) compared to a healthy subject (bottom), suggesting for the presence of an intragenic, heterozygous genomic deletion. Reads were visualized using Integrative Genomics Viewer.

**Supplementary Figure S2. Location of Novel Variants in the Genomic Sequence of *IL1RN*.** Upper-case letters indicate nucleotide located in exons, and lower-case letters nucleotides in untranslated regions or in introns. Start codon ATG is highlighted in blue, and stop codon TAG in red. The variant at donor splicing site is highlighted in green, and the intragenic genomic deletion is highlighted in orange.

**Supplementary Figure S3. mRNA transcript generated by the *IL1RN* allele containing the variant at canonical donor splice site.** Overview of DNA, mRNA and protein sequences of wild-type *IL1RN* allele (upper panels) or *IL1RN* allele containing the splice site variant (bottom panels). In the DNA and mRNA sequences, capital black/blue letters indicate nucleotides from exons, lowercase gray letters indicate nucleotides from introns, dashed blue lines indicate the normal mRNA *IL1RN* splicing, and dashed red line the altered mRNA *IL1RN* splicing identified in subject I-2.

**Supplementary Figure S4. mRNA transcript generated by the *IL1RN* allele containing the genomic deletion.** Overview of DNA, mRNA and protein sequences of wild-type *IL1RN* allele (upper panels) or *IL1RN* allele containing the genomic deletion (bottom panels). In the DNA and mRNA sequences, capital black/blue letters indicate nucleotides from exons, lowercase gray letters indicate nucleotides from introns, dashed blue lines indicate the normal mRNA *IL1RN* splicing, and dashed red line the altered mRNA *IL1RN* splicing identified in subject I-1.

**Supplementary Tables**

**Supplementary Table S1. List of analyzed genes associated with autoinflammatory diseases.** Abbreviations: ADA2, adenosin deaminase 2; ARPC1B, actin related protein C1B; NOCARH, Neonatal-onset cytopenia, autoinflammation, rash and hemophagocytic lymphohistiocytosis; IL-10, interleukin-10; DIRA, deficiency of interleukin-1 receptor antagonist; DITRA, deficiency of interleukin-36 receptor antagonist; FMF, familial Mediterranean Fever; PAAND, Pyrin-associated autoinflammation with neutrophilic dermatosis; MK, mevalonate kinase; AIFEC, Autoinflammation with infantile enterocolitis; NAIAD, NLRP1-associated autoinflammation with arthritis and dyskeratosis; FCAS2, familial cold-induced autoinflammatory syndrome type 2; CAPS, cryopyrin-associated periodic syndromes; PLAID, PLCG2-associated antibody deficiency, and immune dysregulation; APLAID, Autoinflammation and PLCG2-associated antibody deficiency, and immune dysregulation; CANDLE, Chronic atypical neutrophilic dermatosis with lipodystrophy and elevated temperature; PRAAS, Proteasome-associated autoinflammatory syndrome; PAPA, Pyogenic arthritis, pyoderma gangrenosum and acne; Hz/Hc, Hyperzincemia and hypercalprotectinemia syndrome; HOIL-1, Heme-oxidized IRP2 ubiquitin ligase 1; CRIA, cleavage-resistant RIPK1-induced autoinflammatory syndrome; HOIP, HOIL-1-interacting protein; SAVI, STING-associated vasculopathy with onset in infancy; TRAPS11, TNFRSF11A-associated periodic syndrome; TRAPS, TNF Receptor I-associated periodic syndrome; SIFD, Sideroblastic anemia, B-cell immunodeficiency, periodic fevers, and developmental delay; PFIT, periodic fever, immunodeficiency, and thrombocytopenia syndrome.

| **Gene** | **Disease** | **Ref Seq** |  | **Gene** | **Disease** | **Ref Seq** |
| --- | --- | --- | --- | --- | --- | --- |
| *ADA2* | ADA2 Deficiency | NM_001282225.1 |  | *PLCG2* | PLAID-APLAID | NM_002661.3 |
| *ADAR* | Aicardi-Goutières syndrome Type 6 | NM_001111.5 |  | *POMP* | CANDLE/PRAAS | NM_015932.5 |
| *AP1S3* | Pustular Psoriasis | NM_001039569.1 |  | *PSMA3* | CANDLE/PRAAS | NM_002788.3 |
| *ARPC1B* | ARPC1B Deficiency | NM_005720.4 |  | *PSMB4* | CANDLE/PRAAS | NM_002796.2 |
| *CARD14* | Pustular Psoriasis | NM_024110.4 |  | *PSMB8* | CANDLE/PRAAS | NM_148919.3 |
| *CDC42* | NOCARH | NM_001791.4 |  | *PSMB9* | CANDLE/PRAAS | NM_002800.4 |
| *IFIH1* | Aicardi-Goutières syndrome Type 7 | NM_022168.3 |  | *PSMG2* | CANDLE/PRAAS | NM_020232.4 |
| *IL10* | IL-10 Deficiency | NM_000572.2 |  | *PSTPIP1* | PAPA-Hz/Hc | NM_003978.3 |
| *IL10RA* | IL-10R1 Deficiency | NM_001558.3 |  | *RBCK1* | HOIL-1 Deficiency | NM_031229.3 |
| *IL10RB* | IL-10R2 Deficiency | NM_000628.4 |  | *RELA* | Rel-A Haploinsufficiency | NM_021975.3 |
| *IL1RN* | DIRA | NM_173842.2 |  | *RIPK1* | CRIA | NM_003804.5 |
| *IL36RN* | DITRA | NM_173170.1 |  | *RNASEH2A* | Aicardi-Goutières syndrome Type 4 | NM_006397.2 |
| *LACC1* | Lacasse Deficiency | NM_153218.3 |  | *RNASEH2B* | Aicardi-Goutières syndrome Type 2 | NM_024570.3 |
| *LPIN2* | Majeed syndrome | NM_014646.2 |  | *RNASEH2C* | Aicardi-Goutières syndrome Type 3 | NM_032193.3 |
| *MEFV* | FMF-PAAND | NM_000243.2 |  | *RNF31* | HOIP Deficiency | NM_017999.4 |
| *MVK* | MK Deficiencies | NM_000431.3 |  | *SAMHD1* | Aicardi-Goutières syndrome Type 5 | NM_015474.3 |
| *NCSTN* | Hidradenitis suppurativa | NM_015331.2 |  | *TMEM173* | SAVI | NM_198282.3 |
| *NLRC4* | AIFEC | NM_021209.4 |  | *TNFAIP3* | A20 Haploinsufficiency | NM_006290.3 |
| *NLRP1* | NAIAD | NM_033004.3 |  | *TNFRSF11A* | TRAPS11 | NM_003839.3 |
| *NLRP12* | FCAS2 | NM_144687.2 |  | *TNFRSF1A* | TRAPS | NM_001065.3 |
| *NLRP3* | CAPS | NM_001243133.1 |  | *TREX1* | Aicardi-Goutières syndrome Type 1 | NM_033629.5 |
| *NOD2* | Blau syndrome | NM_022162.2 |  | *TRNT1* | SIFD | NM_182916.2 |
| *OTULIN* | Otulipenia | NM_138348.5 |  | *WDR1* | PFIT | NM_017491.4 |

**Supplementary Table S2. Analysis of novel *IL1RN* Variants.** ^1^RefSeq *IL1RN*: NM_173842.3. ^2^Classification of pathogenicity of gene variants performed on the basis of standards and guidelines proposed in the consensus recommendations of the American College of Medical Genetics and Genomics (ACMG) and the Association for Molecular Pathology (AMP). Abbreviations: Chr, chromosome; NHLBI-ESP, National Heart, Lung and Blood Institute-Exome Sequencing Project; gnomAD, Genome Aggregation Database; CSVS, Collaborative Spanish Variant Server; pred, prediction; GERP, Genomic Evolutionary Rate Profiling; CADD, Combined Annotation Dependent Depletion; n.r., not registered; n.a., not applicable.

| **Structural Features of Variants** | | |
| --- | --- | --- |
| Chromosome position (GRCh37) | Chr2: 113,888,736 | Chr2: 113,886091_113,888,684 |
| Chromosome position (GRCh38) | Chr2: 113,131,159 | Chr2: 113,128,514_ 113,131,107 |
| Reference allele | T | - |
| Variant allele | G | Δ2593 bp |
| Gene^1^ | *IL1RN* | *IL1RN* |
| Exon \| Intron | Intron 3 | Intron 1 to exon 3 |
| cDNA alteration | c.318+2T>G | c.116+774del2593bp |
| **Population Genetics (Minor Allele Frequency)** | | |
| 1000 Genomes Project Phase 3 (2015 release) | 0 | 0 |
| NHLBI-ESP (ESP6500SI-V2 version) | 0 | 0 |
| Kaviar database (160204 version) | 0 | 0 |
| gnomAD (v4.0) | 0.000001254 | 0 |
| CSVS (3.0.1. version; February 2021 release) | 0 | 0 |
| **Bioinformatics** | | |
| Mutation Taster_pred | Disease causing | Disease causing |
| SpliceAI_Δscore | 1.00 (Donor loss) | n.a. |
| Database Splicing Consensus Single Nucleotide Variant (dbscSNV) | Pathogenic (score 0.9999) | n.a. |
| GERP (Score) | 5.19 | n.a. |
| Vertebrate PhastCons 100way conservation score | 1.000 | n.a. |
| Vertebrate PhyloP100way conservation score | 3.865 | n.a. |
| CADD phred | 33 | n.a. |
| **Phenotype-Genotype Databases** | | |
| ClinVar database | n.r. | n.r. |
| INFEVERS database | Likely Pathogenic | Likely Pathogenic |
| **ACMG/AMP Variant Classification^2^** | Pathogenic | Pathogenic |
