# Supplementary figures and images for: Case report: Novel compound heterozygous IL1RN mutations as the likely cause of a lethal form of deficiency of interleukin-1 receptor antagonist
Source: Front Immunol. 2024 Apr 5;15:1381447. doi: 10.3389/fimmu.2024.1381447 (PMC11026629; doi:10.3389/fimmu.2024.1381447)

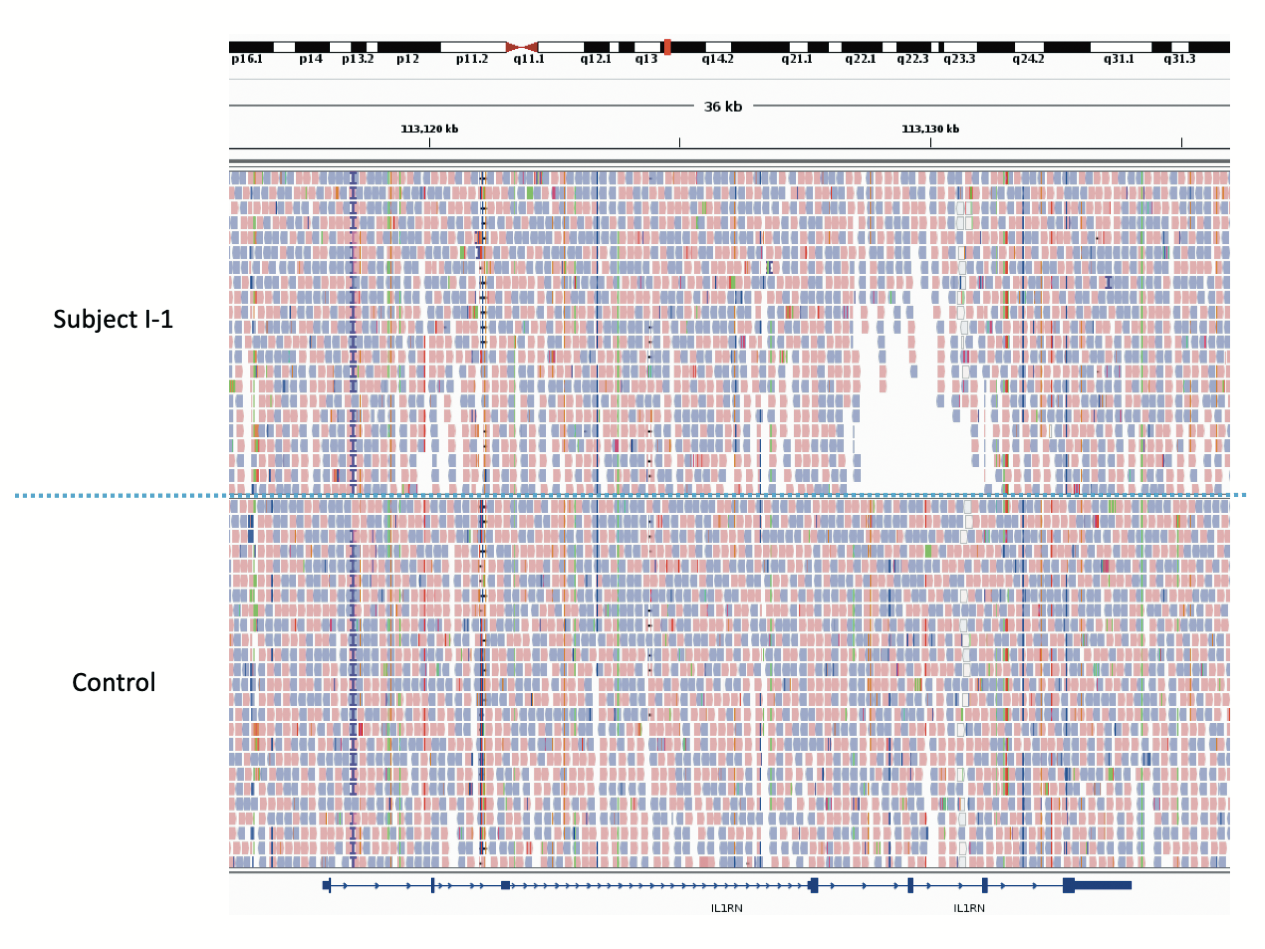

Supplement: Supplementary file 2 [file Image_1.tiff]

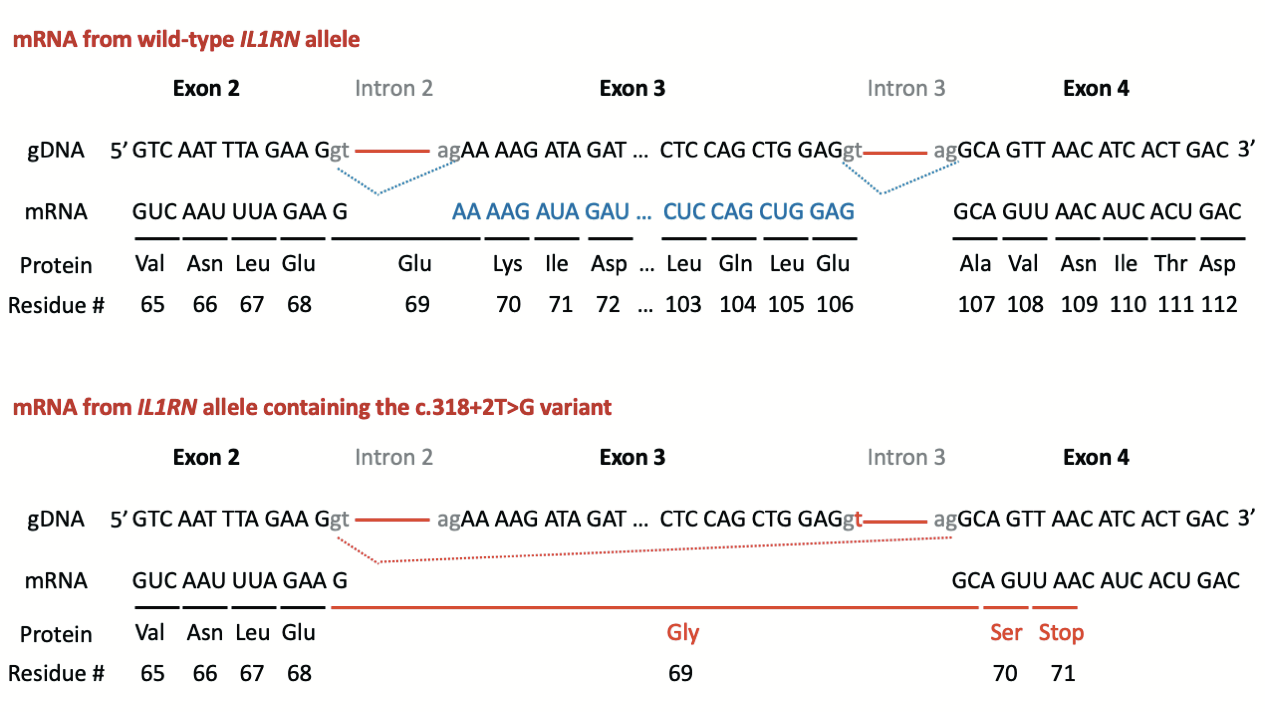

Supplement: Supplementary file 4 [file Image_3.tiff]

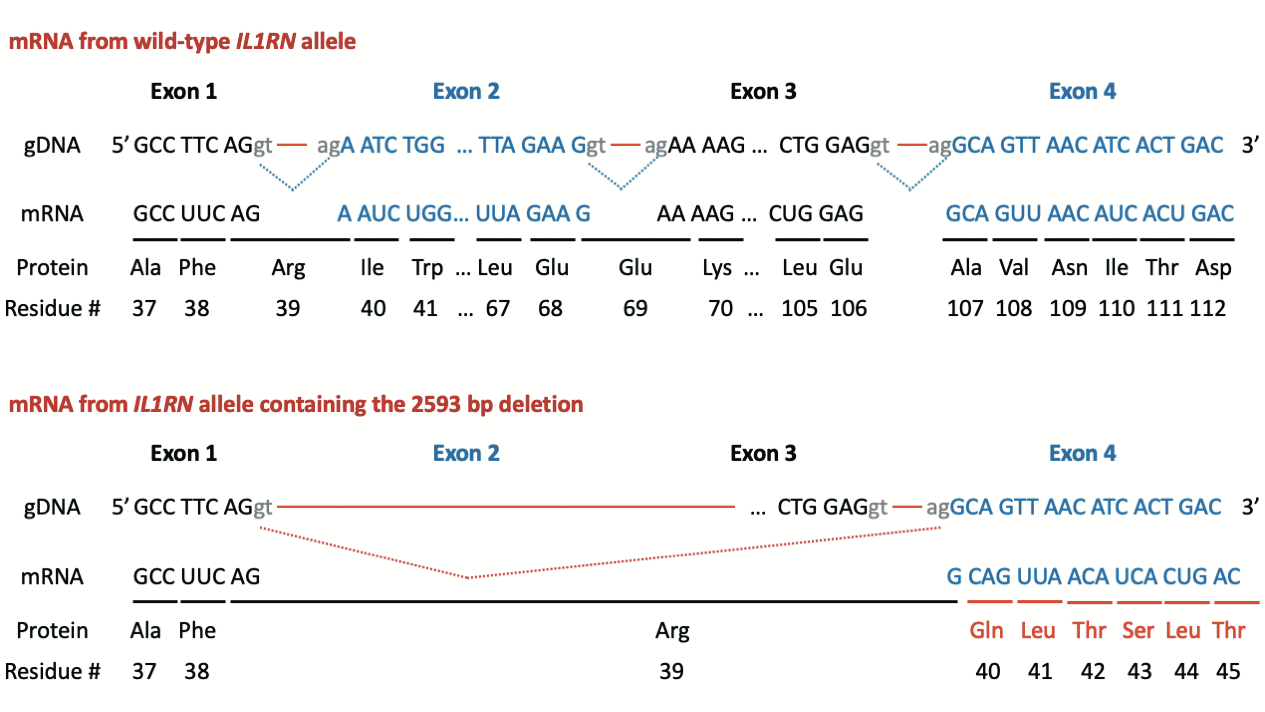

Supplement: Supplementary file 5 [file Image_4.tiff]
