# Supplementary material for: Case report: Novel compound heterozygous IL1RN mutations as the likely cause of a lethal form of deficiency of interleukin-1 receptor antagonist
Source: Front Immunol. 2024 Apr 5;15:1381447. doi: 10.3389/fimmu.2024.1381447 (PMC11026629; doi:10.3389/fimmu.2024.1381447)
Supplement: Supplementary file 3 [file Image_2.pdf]

***IL1RN* Gene (Transcript IL1RN-205 ENST00000409930.3)**

cactccattgcgacacttagtggggttgaaagtgacaacagcaagggtttctcttttggaaatgcgaggagggtatttccgcttctcgcagtggggcagggtggcaga
cgcctagcttgggtgagtgactATTTCTTTATAAACCACAACTCTGGGCCCGCAATGGCAGTCCACTGCCTTGCTGCAGTCACAGAATGG
AAATCTGCAGAGGCCTCCGCAGTCACCTAATCACTCTCCTCCTCTTCCTGTTCCATTCAGAGACGATCTGCCGACCCTCTGGGAG
AAAATCCAGCAAGATGCAAGCCTTCAGgtaaggctaccccaaggaggagaaggtgagggtggatcagctggagactggaaacatatcacagctgccag
gggctgccaggccccagagggcctgagaactgggtttgggctggagaggatgtccattattcaagaaagaggctgttacatgcatgggcttcaggacttgtgtttcaaa
atatcccagatgtggatagtgcgaccggagggctgtcttactttcccagagactcaggaacccagtgagtaatagatgcatgccaaggagtgggactgcgattcaggc
ctagttgaatgtgctgacagagaagcagagaggggcaccaggggcacagcccgaaggcccagactgatatgggcaaggcctgtctgtgctgacatgtcggagggtc
ccactctccagggaccttggtttccccgtctgtgacatctgtgacatgagagtcacgataactccttgtgtgccttacagggttgttgtgaaaattaaatgcacagataat
agcgtaacagtattccgtgcattgtaaagagcctgaaaaccattatgatttgaaaatggaatcggctttgtgagaccatcactattgtaaagatgtgatgctgatagaa
atgacaggactgcttgtgcatgccctctgcagtgtgacattccagcagtgaaatcatgttggggtgacttctcccccactctgacctttatgtttgtctgggccgaggctgc
aagtcgggctctgtgggtgtatgagtgacaagtctctcccttccagatatggggactgtctgcttccctaggttgcctctccctgctctgatcagctagaagctccaggag
atcctcctggaggccccagcaggtgatgtttatccctccagactgaggctaaatctagaaactaggataatcacaaacaggccaatgctgccatatgcaaagcactttg
gtttgcctggccacccctcgtcgagcatgtgggctcttcagagccacctgatgaggtgggtacagttagccacacttcacaggtgaagaggtgaggcacaggtcccag
gtcaggctggccagagctctgtttattacgtctcacagctttgagtcctgctctcaaccagagaggccctttaccaagaagaaaggattgggacccagaatcaggtcac
tggctgaggtagagaggaagccgggttgttcccaagggtagctgctcctgcaggactctgagcaggtcaccagctaatggaggaaaggctctagggaaagacccttc
tggtctcagactcagagcgagttagctgcaaggtgttccgtctcttgaaacttctacctaggtgctatggtagccactagtctcaggtggctatttaaatttatacttaaat
gaatgaaaatagaagaaaatttaaaatccagacccttggtcacactatccacatttaaagaggtcaatagccacatgtggttagtggccaccctattgggcagtgcag
ctacagaacatttttgcatcccagaaagttcttttggatgttgctgctctacagcatgctttgctgaaacagaagtgccttccctgggaatctcagatgggaagcaagtaa
ggaggggagtcaaatgtgggctcactgctcaccagctgtgagggttgggcctgcctcttaaccattgtcagcctcagtcttctcatccatgcatgccgtgggtatactaa
aatactataccccctggaagagctggatgcaaatttgacaagttctgggggacacaggaaggtgccaagcacaaggctgggcacatggtggctgtgcactacagctga
gtccttttccttttcagAATCTGGGATGTTAACCAGAAGACCTTCTATCTGAGGAACAACCAACTAGTTGCTGGATACTTGCAAGGACC
AAATGTCAATTTAGAAGgtgagtggttgccaggaaagccaatgtatgtgggcatcacgtcactttgcccgtctgtctgcagcagcatggcctgcctgcacaaac
cctaggtgcaatgtcctaatccttgttgggtctttgtattcaagtttgaagctgggagggcctggctactgaagggcacatatgagggcagcctgaagagggtgtggag
aggtagagtctaggtcagaggtcagtgcctataggcacagtggtcccagggccacagctgggaagggcaaataccagaaggcaaggttgaccattcccttcctcaag
tgcctattaaggctccatgttcctatgttgttcaaaccctaactcaatcccaaattaatccaccatgtataaggttgagctatgtctcttattcctggacaccatactcagc
catattctggtccacacattaaacaagctggatgaccttgaagaagcttcacccactctgttcctcagctttcccttcagtgggatgatatcaactggacaacaggatgt
gcgattcttttagttccagccttccaggatgttttcactcccctgtttgttgttgtaggatggtattacctccaccttcccaccttccctatgccctggttctgtctcctgtgcct
cgctctgaaagtggatgagacctacaattcctgtcctggtagttctcctaatgaacacactgaagcacgaggaagctgagatttttgttgctacatgagagcatggagg
cctcttagggagagaggaggttcagagactcctaggctcctgtggagccccactcatggccttgttcattttccctgcccctcagcaacactcctattgacctggagcac
aggtatcctggggaaagtgagggaaatatggacatcacatggaacaacatccaggagactcaggcctctaggagtaactgggtagtgtgcatcctggggaaagtga
gggaaatatggacatcacatggaacaacatccaggagactcaggcctctaggagtaactgggtagtgtgcatcctggggaaagtgagggaaatatggacatcacat
ggaacaacatccaggagactcaggcctctaggagtaactgggtagtgtgcatcctggggaaagtgagggaaatatggacatcacatggaacaacatccaggagact
caggcctctaggagtaactgggtagtgtgcttggtttaatcttctatttacctgcagaccaggaagatgagacctctctgcccttctgacctcgggattttagttttgtggg
gaccaggggagatagaaaaatacccggggtctcttcattattgctgcttcctcttctattaacctgaccctcccctctgttcttccccagAAAAGATAGATGTGGT
ACCCATTGAGCCTCATGCTCTGTTCTTGGGAATCCATGGAGGGAAGATGTGCCTGTCCTGTGTCAAGTCTGGTGATGAGACCAG
ACTCCAGCTGGAGgtaaaaacatgctttggatctcaaatcaccccaaaacccagtggcttgaaacaaccaaaatttttcttatgattctgtgggttgaccaggat
tagctgggtagttctgttccatgtggtggaacatgctggggtcactttggaagctgcattcagcagagtgcctggcttgcgctgggcatccaaggtggtccctcatcctcc
aggctctctttccatgtgatctctcagtgtttaagagttagttggagcttccttacagcatggcggctgacttccaaaagggattattccaaaaagagcctcaacatgcag
gcgcttattatgacttctgcttgcatcatcctattggccaaagccagtcacgtggctaagtctagccccctgtgagaggagactgcataagagtgtgaacaccaggaga
cacggtcactgggggccaccactgtaaccatctaccacaggacctgaatctctgtgtgctactcccttgctcaagggccccctacccacgcagacctgctgtcttctag
caaagcccatcctcaggacctttctcttccaatccttattgactcaaattgattagttggtgctccacccagagccctgtgctcctttatctcatgtaatgttaatgggtttcc
cagccctgggaaaacatggctttgtctcaggggcttgctggatgcaagcttaacctcaatgtgagtggccatactgtggcactgtcccatccctcaccagggacactgtt
ctggagggtgactgcctgttctgtgaggagtggggatggctaggacattgcatggaacacaccaccaccccatcttctcagagctcaaaccctgacagaacaccagct
ccacaggccttggcttctgctgatggtgccgtgtatttaccagacttagtggtccaaggccagagtggccagatttcccaaagtcaaggtgtgacagtgggacagcctct
ttgtgtctttgctgtcctaagaaacctgggccaggccaggcgcagtggctcacgcctgtaatcccagcactttgagaagccaaggtgggcagatcacgaggtcaggag
tttgagaccagcctggccaacatggtgaaaccctgtctctattaaaaatagaaaacattagacaggtgtggtggtgcatgcctgtaatcccagctactcaggaggctga
ggcaggagaatcgcttgaacccaggaggtggaggttgcagtgagccgagattgtgccactgcactccagcctaggcgacagagcaagactccgtctcgggaaaatta
attaataaataaataaacctaggtcccagagtcccacagaatggcagacaggagcacctgggggcttttagggtatggcatttcccctgtactaactctgggctgtcca
gagggccatttcatggcgtggagtggagagggaggcagcacaggacttcctaggcctcagctctcacctgcccatcttttgatttccagGCAGTTAACATCACTG
ACCTGAGCGAGAACAGAAAGCAGGACAAGCGCTTCGCCTTCATCCGCTCAGACAGTGGCCCCACCACCAGTTTTGAGTCTGCC
GCCTGCCCCGGTTGGTTCCTCTGCACAGCGATGGAAGCTGACCAGCCCGTCAGCCTCACCAATATGCCTGACGAAGGCGTCAT
GGTCACCAAATTCTACTTCCAGGAGGACGAGTAGTACTGCCCAGGCCTGCCTGTTCCCATTCTTGCATGGCAAGGACTGCAGGG
ACTGCCAGTCCCCCTGCCCCAGGGCTCCCGGCTATGGGGGCACTGAGGACCAGCCATTGAGGGGTGGACCCTCAGAAGGCGT
CACAACAACCTGGTCACAGGACTCTGCCTCCTCTTCAACTGACCAGCCTCCATGCTGCCTCCAGAATGGTCTTTCTAATGTGTGA
ATCAGAGCACAGCAGCCCCTGCACAAAGCCCTTccatgtcgcctctgcattcaggatcaaaccccgaccacctgcccaacctgctctcctcttgccactg
